# Clinical characteristics and prognosis of pulmonary renal syndrome in West China

**DOI:** 10.1038/s41598-023-27559-7

**Published:** 2023-01-09

**Authors:** Maozhi Tang, Jun Zhang, Xiaosong Xu, Qianguang Pan, Hongwen Zhao

**Affiliations:** 1 Urinary Nephropathy Center, The Second Affiliated Hospital of Chongqing Medical University, Chongqing, 400010 China; 2 Department of Kidney, The First Affiliated Hospital of Army Medical University, Gaotanyan Zhengjie, Shapingba District, Chongqing, 400038 China

**Keywords:** Diseases, Nephrology

## Abstract

Pulmonary renal syndrome (PRS) is a rare and life-threatening syndrome. Interstitial lung disease (ILD) has been recently considered another phenotype of lung dysfunction in patients with PRS, but there are very limited data. The characteristics of fifty PRS patients were retrospectively reviewed after a 3-year follow-up, and the differences between PRS patients whose lung dysfunction presented as diffuse alveolar hemorrhage (DAH group) and those with interstitial lung disease (ILD group) were also analyzed. The median age at diagnosis of PRS patients was 50.78 ± 17.88 years, and the main symptoms at disease onset were proteinuria (94.00%), hemoptysis (68.00%), dyspnea (32.00%) and fever (12.00%). DAH patients were younger and had significantly lower hemoglobin levels, a higher incidence of hemoptysis, and higher serum creatinine levels at onset than ILD patients. Univariate analyses of PRS patients showed that respiratory failure and the initiation of mechanical ventilation predicted patient death and that the initiation of hemodialysis and higher serum creatinine levels at onset predicted ESRD. Multivariate analyses showed that respiratory failure and anti-GBM antibody positivity could independently predict patient death. Survival analyses showed that 1- and 3-year patient survival rates and ESRD-free survival rate were not significantly different between the two groups. ILD was another important phenotype of lung dysfunction in patients with PRS. Poor outcomes were observed in PRS patients with ILD and in PRS patients with DAH.

## Introduction

Pulmonary renal syndrome (PRS) is a complex condition with simultaneous involvement of the kidneys and lungs and is caused by autoimmune diseases or presents secondary to infectious diseases. Pulmonary diffused alveolar hemorrhage (DAH) and rapidly progressive glomerulonephritis (RPGN) are the most typical clinical features^1,2^. Corresponding histological features include glomerular crescents on renal biopsy and pulmonary capillaritis on lung biopsy^3^. Anti-neutrophil cytoplasmic antibody (ANCA)-associated vasculitis (ASSV) and anti-glomerular basement membrane (GBM) disease are the most common autoimmune disease causing PRS, but other autoimmune conditions, including systemic lupus erythematosus or anti-phospholipid antibody syndrome, have also been reported to cause PRS^4^. PRS has also been reported to occur secondary to infectious diseases, including methicillin-resistant *Staphylococcus aureus* (MRSA), Legionnaires’ disease and bacterial endocarditis^5–7^. However, it is worth noting more publications have reported an association between interstitial lung disease (ILD) and ASSV. ILD, another phenotype of lung dysfunction in patients with PRS, can seriously affect lung function and reduce long-term survival of PRS patients^8,9^. Unlike diagnosing DAH-related PRS, diagnosing ILD-related PRS may be more complicated because of its subacute disease onset and progression, leading to missed opportunities for early intervention. To date, only small studies with a short follow-up period have reported outcome data for PRS; moreover, the clinical features of PRS patients with ILD-related lung dysfunction have not been well described. Hence, the aim of the present study is to report the outcome of 50 PRS patients who were divided into 2 groups according to lung involvement (a DAH group and an ILD group).

## Methods

This was a retrospective analysis of consecutive patients diagnosed with PRS managed at the First Affiliated Hospital of Army Medical University between January 2013 and January 2018, and patients with a 3-year observation were included in the analysis. The study was approved by the Ethics Committee of the First Affiliated Hospital of Army Medical University (No. KY2020302). The requirement for written informed consent was waived by this ethics committee due to the observational, retrospective nature of this study. We also followed the practice of the Declaration of Helsinki and relevant guidelines, regulations and policies as required by the journal. The inclusion criteria were as follows: (1) lung dysfunction due to DAH or ILD; (2) renal dysfunction caused by GN or with proteinuria; and (3) serological test showing ANCA or anti-GBM antibody positivity. Exclusion criteria were as follows: (1) definite lung infection and (2) lack of follow-up data. Diffuse pulmonary hemorrhage was defined as the presence of diffuse, bilateral, parenchymal infiltrates on chest radiograph or CT scan, and either hemoptysis or direct visualization of bleeding in bronchoscopy. Interstitial lung disease was defined as one or more of the following CT scan features: ground glass opacities, reticular shadowing, interlobular septal thickening, consolidations and honeycombing. Baseline characteristics, clinical, serological and biochemical features, and outcome data concerning patient survival, ESRD-free survival and lung function were recorded.

### Statistical analysis

Data analysis and graphs were performed using Prism 5.0 (GraphPad Software, La Jolla, CA) and Stata version 12.0 (StataCorp LP, College Station, Tex., USA). Column statistics are presented as the mean ± SD, along with the 95% confidence intervals (CI) of the mean and percentage (%). Quantitative data were analyzed with t tests and chi-square tests to compare categorical variables between the groups. Univariate and multivariate conditional logistic regression models were performed to predict death or ESRD. Multivariate models included covariates whose p value was less than 0.20 in the univariate models. The predictors of death or ESRD were reported as unadjusted or adjusted odds ratios (ORs) along with their 95% CIs. The log-rank test was used to ascertain unadjusted survival differences, and they were plotted as Kaplan–Meier curves. The results were considered statistically significant when their p value was less than 0.05.

## Results

### Characteristics of PRS patients

**Figure 1:**
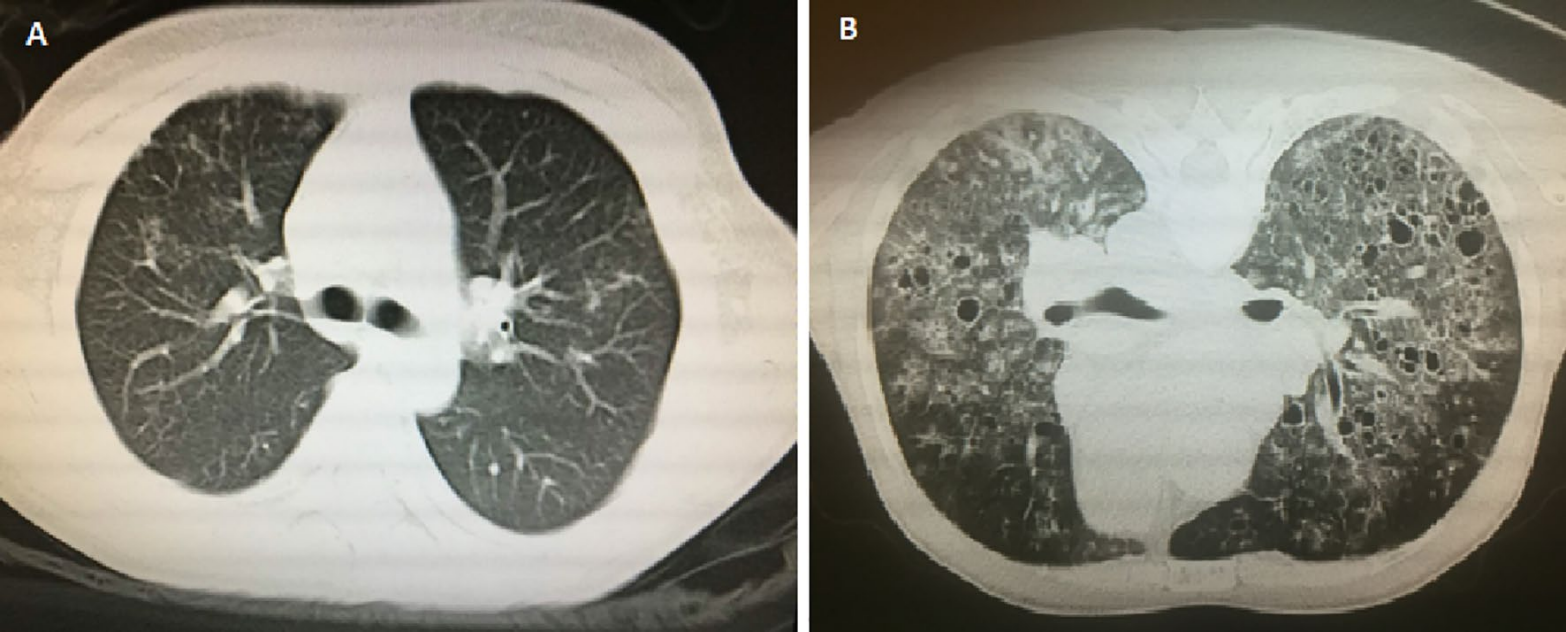
Typical imaging of DAH (**A**) and ILD (**B**) from our PRS patients.

Sixty-three patients met the diagnostic criteria of of PRS and were included; however, 13 patients were excluded due to a lack of survival data, so 50 patients were divided into the DAH group (37 patients) and the ILD group (13 patients) and then finally included in the analysis. The typical imaging findings of DAH and ILD in our PRS patients are presented in Fig. 1. The main clinical, laboratory, pathological, and immunological features are summarized in Table 1. We included 50 patients with a male-to-female ratio of 1.17 (27:23). The median age at diagnosis was 50.78 ± 17.88 years, and 19 (38.00%) were over the age of 60 years. The average duration of prodromal illness was 2.33 ± 2.98 months, and the DAH group had an earlier onset (1.99 ± 2.86 vs. 3.31 ± 3.19 months, p = 0.02). The main symptoms at disease onset were proteinuria (47 cases, 94.00%), hemoptysis (34 cases, 68.00%), dyspnea (16 cases, 32.00%) and fever (6 cases, 12.00%), along with complications including respiratory failure (20 cases, 40.00%), heart failure (8 cases, 16.00%) and pulmonary arterial hypertension (PAH) (14 cases, 28.00%). Among these clinical features, the DAH group had a significantly higher incidence of hemoptysis than the ILD group (78.38% vs. 38.46%, p = 0.014).

**Table 1:** Characteristics of the included 50 PRS patients.

	Overall (n = 50)	DAH (n = 37)	ILD (n = 13)	p value
Age(yr)	50.78 ± 17.88	47.54 ± 19.21	60.00 ± 8.59	0.03
Sex	27 M/23F	19 M/18F	8 M/5F	——
Duration of prodromal illness (months)	2.33 ± 2.98	1.99 ± 2.86	3.31 ± 3.19	0.02
**Clinical features**				
Hemoptysis, n (%)	34 (68.00)	29 (78.38)	5 (38.46)	0.01
Proteinuria, n (%)	47 (94.00)	36 (97.30)	11 (84.62)	–
24-h UAE (mg, n)	2370 ± 1176 (21)	2357 ± 1265 (17)	2425 ± 831.6 (4)	–
Fever, n (%)	6 (12.00)	5 (13.51)	1 (7.69)	–
Dyspnea, n (%)	16 (32.00)	11 (29.73)	5 (38.46)	–
Respiratory failure, n (%)	20 (40.00)	17 (45.95)	3 (23.08)	–
Heart failure, n (%)	8 (16.00)	6 (16.22)	2 (15.38)	–
PAH, n (%)	14 (28.00)	10 (27.03)	4 (30.77)	–
**Laboratory tests**				
WBC (10*9/L)	7.57 ± 2.60	7.80 ± 2.73	6.91 ± 2.14	–
HBG (g/L)	83.78 ± 25.75	80.43 ± 23.05	93.31 ± 31.30	0.16
PLT (10*9/L)	222.70 ± 105.40	222.50 ± 105.00	223.20 ± 111.10	–
CRP (mg/L)	28.32 ± 37.11	30.00 ± 36.16	23.53 ± 40.81	–
PCT (ng/ml)	0.54 ± 1.05	0.62 ± 1.16	0.30 ± 0.62	–
Scr (μmol/L)	484.90 ± 355.00	552.40 ± 374.00	292.60 ± 202.30	0.02
Alb (g/L)	30.43 ± 4.98	29.96 ± 4.31	31.75 ± 6.55	–
C3 (g/L)	0.79 ± 0.16	0.79 ± 0.16	0.80 ± 0.15	–
C4 (g/L)	0.21 ± 0.07	0.21 ± 0.07	0.21 ± 0.07	–
ANCA, n (%)	44 (88.00)	31 (83.78)	13 (100)	–
MPO-ANCA, (n)	33	23	10	–
PR3-ANCA, (n)	11	8	3	–
Anti-GBM, n (%)	9 (18.00)	8 (21.62)	1 (7.69)	–
ANCA + Anti-GBM, n (%)	3 (6.00)	2 (15.38)	1 (7.69)	–
**Glomerular findings (N = 23)**	–	n = 17	n = 6	–
Crescentic glomeruli, % (range)	–	45.22 ± 34.80 (0–100)	56.33 ± 13.16 (44.40–77.80)	–
Sclerotic glomeruli, % (range)	–	24.63 ± 23.40 (0–88.20)	23.55 ± 10.28 (11.80–38.50)	–
Normal glomeruli, % (range)	–	30.04 ± 30.35 (0–100)	20.12 ± 12.64 (0–35.30)	–
**Treatment**				
ICU stay, n (%)	9 (18.00)	9 (24.32)	0	0.09
Mechanical ventilation, n (%)	9 (18.00)	9 (24.32)	0	0.09
Initial hemodialysis (%)	20 (40.00)	16 (43.24)	4 (30.77)	–
Plasmapheresis, n (%)	29 (58.00)	24 (64.86)	5 (38.46)	–
Corticosteroid pulses, n (%)	39 (78.00)	32 (86.49)	7 (53.85)	–
Corticosteroids, n (%)	49 (98.00)	37 (100)	12 (92.31)	–
Cyclophosphamide, n (%)	41 (82.00)	32 (86.49)	9 (69.23)	–
Rituximab, n (%)	6 (12.00)	4 (10.81)	2 (15.38)	–
Other immunosuppressive agent, n (%)	6 (12.00)	4 (10.81)	2 (15.38)	–
**Outcome**				
Death, n (%)	14 (28.00)	11 (29.73)	3 (23.08)	0.73
ESRD, n (%)	20 (40.00)	15 (40.54)	5 (38.46)	1

*M* male, *F* female, *HBG* hemoglobin, *WBC* white blood cell, *CRP* C reactive protein, *PCT* procalcitonin, *GM* galactomannan, *G test* (1,3)-β-D glucan test, *T-spot* tuberculosis-specific enzyme-linked immunospot assay, *C3* complement 3, *C4* complement 4, *MPO* myeloperoxidase, *PR3* proteinase 3, *ICU* intensive care unit.

Laboratory tests suggested that the DAH group had a significantly higher serum creatinine level than the ILD group (552.40 ± 374.00 vs. 292.60 ± 202.30, p = 0.022); however, the DAH group had a lower HBG level than the ILD group, but no significant difference was found (80.43 ± 23.05 vs. 93.31 ± 31.30, p = 0.16). There was no significant difference between the two groups in terms of 24-h UAE, WBC, HBG, PLT, CRP, PCT, Alb, C3 or C4 levels.

A total of 5.13% of G tests, 6.25% of GM tests and 4.88% of T-spot tests were positive in these patients; however, none of them were verified to have fungal or tuberculosis infections. Among the 50 cases, 44 (88%) cases were determined to be ANCA-related, and 9 (18%) were determined to be anti-GBM antibody-related. Three (6%) patients were positive for both ANCA and anti-GBM antibodies. In 23 renal biopsies, glomerular change mainly manifested as crescentic glomeruli, and the proportions of crescentic glomeruli, sclerotic glomeruli and normal glomeruli were not different between the two groups.

Nine (18.00%) patients with PRS were treated in the ICU under mechanical ventilation. Twenty (40.00%) patients underwent hemodialysis because of rapidly progressive renal failure, and 28 (58.00%) patients received plasmapheresis (PE) therapy. Most patients also received corticosteroids and cyclophosphamide. Six (12.00%) patients received rituximab therapy without severe adverse reactions. The DAH group also had a higher rates of ICU admission, mechanical ventilation and dialysis treatment; however, the differences were not significant.

### Prognostic factors for death

The prognostic factors for death are presented in Table 2. Additionally, unadjusted prognostic factors for death included respiratory failure (6.500 [1.652–25.575]; p = 0.007) and mechanical ventilation (OR: 8.25 [1.688–40.319]; p = 0.009) are presented in Table 2. In multivariate analysis, after adjusting for the factors of respiratory failure, PAH, mechanical ventilation, anti-GBM antibodies, HBG, CRP and ESRD onset to dialysis, factors including respiratory failure (OR: 11.755 [1.130–122.314]; P = 0.039) and anti-GBM antibody positivity (OR: 16.320 [1.192–223.521]) could predict death.

**Table 2:** Prognostic factors for the death of PRS patients.

	Death (unadjusted)			Death (adjusted)		
	OR (95% CI)	SE	*P* value	OR (95% CI)	SE	*P* value
Sex	0.54 (0.15–1.87)	0.34	0.33	–	–	–
Age	0.99 (0.96–1.03)	0.02	0.66	–	–	–
Duration of prodromal illness	1.04 (0.85–1.27)	0.11	0.68	–	–	–
Fever	1.33 (0.22–8.25)	1.24	0.76	–	–	–
Hemoptysis	2.07 (0.49–8.80)	1.53	0.32	–	–	–
Respiratory failure	6.50 (1.65–25.58)	4.54	0.007	11.76 (1.13–122.31)	14.05	0.04
PAH	2.63 (0.70–9.81)	1.77	0.15	2.41 (0.37–15.81)	2.32	0.36
Initial hemodialysis	0.56 (0.15–2.13)	0.38	0.4	–	–	–
Mechanical ventilation	8.25 (1.69–40.32)	6.68	0.009	0.62 (0.04–10.59)	0.9	0.75
ANCA positive	0.33 (0.06–1.90)	0.3	0.22	–	–	–
anti-GBM antibody positive	4.44 (0.98–20.09)	3.42	0.05	16.32 (1.19–223.52)	21.79	0.04
DAH	0.83 (0.21–3.32)	0.59	0.8	–	–	–
ILD	1.20 (0.30–4.79)	0.85	0.8	–	–	–
Alb	1.00 (0.89–1.14)	0.06	0.95	–	–	–
Scr	1 (1.00–1.002)	0.001	0.65	–	–	–
HBG	0.98 (0.95–1.01)	0.02	0.18	0.96 (0.92–1.004)	0.02	0.07
WBC	0.93 (0.72–1.19)	0.12	0.55	–	–	–
CRP	1.02 (1.00–1.04)	0.01	0.06	1.01 (0.99–1.04)	0.01	0.34
ESRD onset to dialysis	0.31 (0.07–1.28)	0.22	0.11	0.15 (0.02–1.29)	0.16	0.08

Variates of p value <0.2 were enrolled in multivariate analysis. Adjusted for respiratory failure, PAH, mechanical ventilation, anti-GBM antibodies, HBG, CRP and ESRD to dialysis.

### Prognostic factors for ESRD

The analysis of prognostic factors for ESRD is displayed in Table 3. Unadjusted prognostic factors for ESRD were calculated, including initial hemodialysis in treatment (OR: 4.929 [1.439–16.884]; p = 0.011) and higher serum creatinine levels at onset (OR: 1.003 [1.001–1.005]; P = 0.012); however, multivariate analysis was not conducted due to a limited amount of significant prognostic factors.

**Table 3:** Prognostic factors for ESRD in PRS patients.

ESRD (unadjusted)			
	OR (95% CI)	SE	*P* value
Sex	1.07 (0.34–3.33)	0.62	0.91
Age	1.00 (0.96–1.03)	0.02	0.76
Duration of prodromal illness	1.11 (0.92–1.35)	0.11	0.28
Fever	0.72 (0.12–4.37)	0.66	0.72
Hemoptysis	1.74 (0.50–6.09)	1.11	0.39
Respiratory failure	0.70 (0.22–2.27)	0.42	0.56
PAH	1.18 (0.34–4.16)	0.75	0.8
Initial hemodialysis	4.93 (1.44–16.88)	3.1	0.01
Mechanical ventilation	0.37 (0.07–1.98)	0.31	0.24
ANCA positive	0.63 (0.11–3.49)	0.55	0.6
anti-GBM antibody positive	2.17 (0.50–9.33)	1.61	0.3
DAH	1.09 (0.30–3.99)	0.72	0.9
ILD	0.92 (0.25–3.35)	0.61	0.9
Alb	1.00 (0.89–1.12)	0.06	0.95
Scr	1.003 (1.001–1.005)	0.001	0.01
HBG	0.99 (0.96–1.01)	0.01	0.26
WBC	0.96 (0.77–1.20)	0.11	0.7
CRP	0.99 (0.97–1.01)	0.01	0.24

### Outcome of PRS patients and survival analysis

After the 3-year follow-up, 14 (28.00%) patients died (11 patients in the DAH group and 3 patients in the ILD group), and 20 (40.00%) patients required regular dialysis (15 patients in the DAH group and 5 patients in the ILD group). The renal progression of 18 surviving nondialysis patients was calculated, and their average serum creatinine increased from 212.3 ± 166.90 μmol/L to 226.8 ± 119.0 μmol/L after 3 years of observation (P = 0.586). In terms of pulmonary involvement, 10 of the 20 respiratory failure patients died (8 in the DAH group, 2 in the ILD group), of whom 2 died of lung function deterioration associated with ILD.

**Figure 2:**
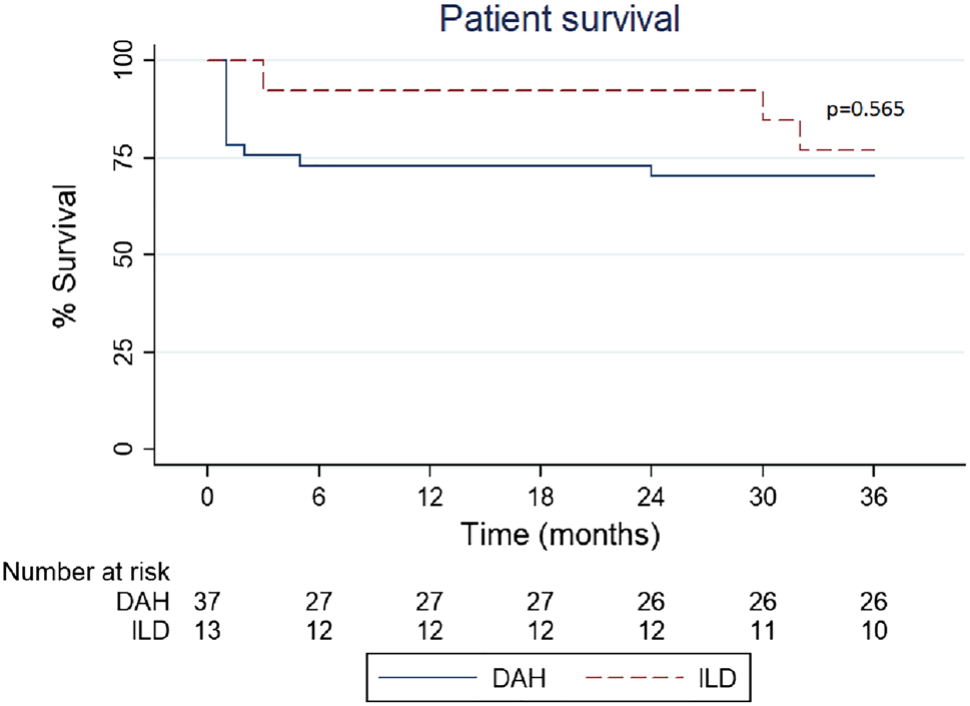
Comparison of patient survival between the DAH and ILD groups in the 3-year observation.

**Figure 3:**
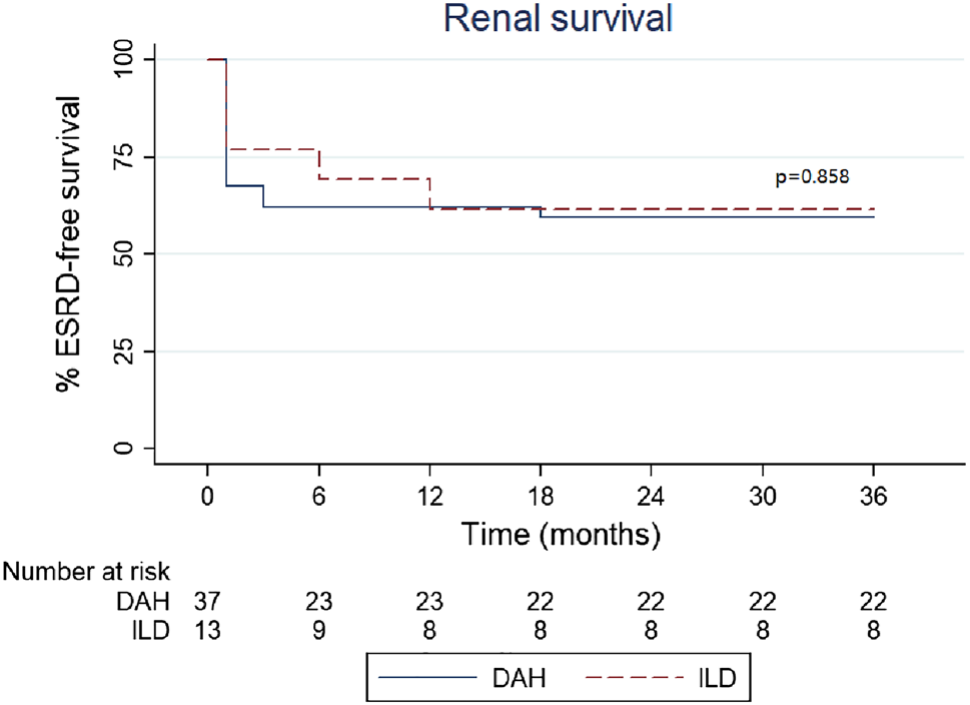
Comparison of renal survival between the DAH and ILD groups in the 3-year observation.

In survival analyses, the 1-year patient survival rates were 72.97% in the DAH group and 92.31% in the ILD group. The 3-year patient survival rates were 70.27% in the DAH group and 76.92% in the ILD group. However, there were no significant differences between the two groups in terms of 1-year and 3-year patient survival rates (see Fig. 2, P > 0.05). The 1-year ESRD-free survival rates were 62.16% in the DAH group and 61.54% in the ILD group. The 3-year ESRD-free survival rates were 59.46% in the DAH group and 61.54% in the ILD group. The 1-year and 3-year ESRD-free survival rates between the two groups were not significantly different (see Fig. 3, P > 0.05).

## Discussion

To date, most published studies on PRS have been retrospective studies with small samples, and the clinical features, prognostic factors for death and ESRD and patient or ESRD-free survival rates have not been well described. Previous studies have not considered PRS cases with pulmonary involvement presenting as ILD. To our knowledge, this retrospective study of PRS has the largest sample size to date.

Previous studies have reported that PRS has a mortality rate that ranges from 29.4% to 50%^10–12^. Here, we report a similar mortality of 28.57% and first reported an overall 3-year patient survival rate of 77.78%. ILD is an uncommon complication of ANCA vasculitis and is usually observed in patients older than 60 years, occurring more frequently in Asian patients than in European patients^13^. Most often, pulmonary fibrosis develops before or coincides with the onset of systemic vasculitis symptoms. This pulmonary pathologic change might be corrected by timely therapy with corticosteroids, cyclophosphamide or mycophenolate mofetil at an early stage; otherwise, systemic vasculitis can lead to irreversible lung damage. Comarmond et al. followed a series of 49 ANCA-related ILD patients for 48 months; however, 11 of the 18 patients died and their deaths were directly associated with chronic lung function deterioration^14^. In our study, 2 patients died of ILD-associated chronic respiratory failure in the 3-year observation, suggesting that the evaluation and maintenance of pulmonary function in patients with this characteristic have considerable value in improving the prognosis.

Consistent with other previous studies, our ANCA-associated vasculitis patients had a median age at diagnosis of 50.78 ± 17.88, ANCA-associated vasculitis was more prevalent in older patients^15^, and anti-GBM antibody-positive patients were relatively younger (40–50)^16^. Moreover, in our study, the DAH group had a younger age distribution than the ILD group. Our data suggested that the DAH group had a higher incidence of hemoptysis, respiratory failure, mechanical ventilation use and ICU stay, accompanied by more severe anemia and kidney damage. However, the survival rate of patients in the DAH group was not significantly lower than that of patients in the ILD group, reflecting a better therapeutic effect in DAH patients, which might be associated with the use of a high dose of corticosteroids and that 64.86% of them received plasmapheresis therapy.

PAH is a severe condition characterized by chronic obstruction of small pulmonary arteries leading to progressive right heart failure and, ultimately, death. PAH is associated with poor survival rates and is commonly reported in patients with autoimmune diseases such as ANCA-associated vasculitis, systemic sclerosis (SSc), anti-GBM disease and SLE^17–19^. Twenty-eight percent of our patients were diagnosed with PAH; however, the univariate logistic regression analysis suggested that PAH was not a significant predictive factor for death or ESRD (P = 0.358, P = 0.797). Anti-GBM antibody-associated PRS and respiratory failure were the leading causes of death in PRS patients. Generally, compared to ANCA-positive patients, alveolar hemorrhage and hypoxemia were more severe in anti-GBM antibody-positive patients; hence, lifesaving therapy including methylprednisolone and sufficient plasmapheresis should be initiated at an early stage. Unfortunately, similar to Gallagher’s report^20^, the mean duration of prodromal illness was as long as 2.33 ± 2.98 months, and renal or lung involvement might cause chronic and irreversible damage in this long time period. As described in our study, a high serum creatinine level was consistent with severe renal damage and predicted ESRD. Hence, the key takeaway we drew from this analysis was the importance of making an early diagnosis and providing timely and effective treatments.

Early aggressive therapy could certainly improve the outcomes of PRS; however, the associated economic factors might negatively affect our current practice because of the high costs of therapy, such as plasmapheresis, ventilator use, and monoclonal antibody therapy. The currently used induction treatment (cyclophosphamide with high-dose corticosteroids) has significantly improved the outcome of AAV but is associated with high toxicity and infectious complications^21^. Plasmapheresis is a lifesaving therapy in PRS with ANCA-related SVV and DAH^22^ or rapidly progressive renal failure (Scr > 500 µmol/L)^23,24^. Plasmapheresis should be recommended in AAV and anti-GBM disease therapy to clear circulating antibodies with a low risk of infection. Monoclonal antibody therapy, such as rituximab, alemtuzumab and tocilizumab, has been used to treat vasculitis and seems more effective than traditional protocols^25,26^. A recent study that explored pulmonary pathological changes in ILD patients demonstrated follicular B-cell hyperplasia and interstitial plasma cell infiltration, suggesting that B-cell inhibition was a potential therapeutic method^27–29^ and that monoclonal antibody therapy should be recommended. Another study found that alternative complement pathway activation participates in the pathogenesis of AAV. Avacopan is a selective inhibitor of the C5a receptor. Avacopan has been shown to have at least similar efficacy to high-dose corticosteroids in active AAV patients with renal involvement, but no major safety issues have been reported. Avacopan has the potential to replace corticosteroids in induction and maintenance treatment, improving patient outcomes by specific targeted therapy and reducing corticosteroid-related side effects^30^.

In conclusion, concerning these limited data, both ILD and DAH were phenotypes of PRS. Both groups had poor prognoses and needed more aggressive therapy. Hence, it is crucial to diagnose patients and identify prognostic factors in the early period before disease progression acceleration. Our paper has two major shortcomings. First, it is a retrospective small-sample cohort study with an insufficient follow-up time. Second, the prevalence of autoimmune diseases is closely related to race, but we only studied local populations in China, thus the sample representativeness is limited. Therefore, a multicenter, prospective and large-sample cohort study is needed.

## Data Availability

The datasets generated and/or analyzed during the current study are available from the corresponding author on reasonable request.

## Abbreviations

PRS: Pulmonary renal syndrome
DAH: Diffuse alveolar hemorrhage
ILD: Interstitial lung disease
GBM: Glomerular basement membrane
ESRD: End-stage renal disease
RPGN: Rapidly progressive glomerulonephritis
ANCA: Antineutrophil cytoplasm antibodies
GN: Glomerulonephritis
PAH: Pulmonary arterial hypertension
PE: Plasmapheresis
UAE: Urinary albumin excretion
